# Molecular diagnosis and genotyping of *Orientia tsutsugamushi* in Maesot and Chiangrai, Thailand

**DOI:** 10.3389/fitd.2023.1146138

**Published:** 2023-05-15

**Authors:** Artharee Rungrojn, Elizabeth M. Batty, Carlo Perrone, Mohammad Yazid Abdad, Tri Wangrangsimakul, Tobias Brummaier, Rose McGready, Nicholas P. J. Day, Stuart D. Blacksell

**Affiliations:** 1 Mahidol Oxford Tropical Medicine Research Unit, Faculty of Tropical Medicine, Mahidol University, Bangkok, Thailand; 2 Centre for Tropical Medicine and Global Health, Nuffield Department of Clinical Medicine, University of Oxford, Oxford, United Kingdom; 3 Shoklo Malaria Research Unit, Mahidol-Oxford Tropical Medicine Research Unit, Faculty of Tropical Medicine, Mahidol University, Mae Sot, Thailand; 4 Swiss Tropical and Public Health Institute, Allschwil, Switzerland; 5 University of Basel, Basel, Switzerland

**Keywords:** *Orientia tsutsugamushi*, scrub typhus, northern Thailand, acute undifferentiated fever (AUF) patients, eschar

## Abstract

**Introduction:**

Scrub typhus is a neglected tropical disease with an estimated 1 million cases annually. The Asia-Pacific region is an endemic area for scrub typhus, especially in Thailand.

**Methods:**

Between June 2018 and December 2019, 31 patients with acute undifferentiated febrile illness (AUFI) were recruited for clinical trials and tested positive by a scrub typhus IgM RDT.

**Results:**

Of the 17 buffy coat patient samples tested by 47kDa real-time PCR and 56kDa type-specific antigen (TSA) nested PCR, 94% (16/17) were positive, and of the 11 patients that presented with eschar lesions, 100% (11/11) of the eschar samples were confirmed positive. Genetic analysis of the 560 bp partial 56-kDa TSA gene demonstrated that most Orientia tsutsugamushi (Ot) infections were with Karp, Gilliam, Taiwan, P23, and CM606-like strains.

**Discussion:**

This is the second occasion that the CM606-like and P23-like strains were reported in northern Thailand (first reported in 2011 and 2013, respectively). This study demonstrates that 1) the eschar remains the most reliable biological sample for PCR diagnosis of scrub typhus and 2) Northwestern Thailand has significant diversity of Ot strains, which underlines the requirement for ongoing surveillance to increase our understanding of Ot diversity to ensure accurate diagnostics and treatment.

## 1 Introduction

*Orientia tsutsugamushi* (Ot) is a Gram-negative obligate intracellular bacteria belonging to the family Rickettsiaceae, related to the genus *Rickettsia* (1). Scrub typhus is a mite-borne disease caused by Ot, with small mammals, especially rodents, serving as hosts and possibly reservoirs (2–4). Patients are infected through the bite of infected larval stage trombiculid mites (5). Ot is maintained in trombiculid mites populations *via* transovarial and transstadial transmission (1). Scrub typhus, similar to other causes of acute undifferentiated fever (AUF), typically presents with non-specific symptoms such as headache, fever, rash, and myalgia (6, 7). The presence of an eschar (necrotic tissue at the bite site) is an important clinical sign of scrub typhus infection, though not always present (7, 8). Delayed diagnosis and treatment can lead to severe organ failure, and median untreated mortality has been estimated to be approximately 6%; however, mortality ranges widely between 0-70%, depending on the region and accessibility to appropriate diagnostics and treatment regimens (8). Patient outcomes following scrub typhus infection can be improved by prompt diagnosis through clinical symptoms such as eschar with febrile illness, followed by prompt treatment (8). Existing serological methods used for scrub typhus diagnosis, include indirect immunofluorescence assay (IFA), ELISA, and RDT (rapid diagnostic tests), have shortcomings that may result in false positives (9–11). In the early stages of the disease, molecular assays can be used as an alternative to the diagnostic serological gold standard (IFA) (9, 12–14). Sequencing of Ot genes isolated from patients for diagnostic or research purposes has been used to understand better the epidemiology and distribution of Ot strains (15, 16).

The 56-kDa type-specific antigen gene (TSA) is a practical molecular diagnostic target for Ot (17). The 56-kDa TSA gene has an open reading frame (ORF) of approximately 1600 bp in length, encoding around 530 amino acids. The protein it encodes is located on the Ot outer membrane. The 56-kDa TSA is the primary immunogen responsible for eliciting neutralizing antibodies and acts as adhesin and invasin to the host cells (18, 19).

Scrub typhus is caused by various species e.g., *O. chuto, O. chilonensis*, other than *O. tsutsugamushi* has recently been reported to be more globally distributed than previously recognized (6, 20). Endemic areas of scrub typhus transmission include rural areas where many Southeast Asia residents reside (21–24). More than a billion people are at risk of infection, and about 1 million cases are thought to occur annually in the region (1). The reported incidence of the disease in Thailand has increased dramatically over the past two decades (25) due to various causes such as climate change, population expansion into uninhabited rural areas, better diagnostics, and public health awareness (25).

*Orientia tsutsugamushi* is widely distributed in Thailand. Ruangareerate et al. studied acute fever of unknown origin (FUO) in six provinces in Thailand’s northeastern, northern, southern, and central regions; antibodies against Ot were detected in 4.3-4.8% of FUO patients, and 7.95% were PCR positive for Ot DNA of these samples (26). Antibodies against Ot were detected in human samples from Chonburi province in eastern Thailand; Karp, Kato, and Gilliam genotype strains were reported from the rodent and chigger samples (27). Rodkvamtook et al. reported three genotypes (related to Gilliam, LA and TA strains) in blood samples from 26 scrub typhus-infected children in Ban Pongyeang, northern Thailand (5). The national disease surveillance system data reported 103,345 cases of scrub typhus between 2003–2018. The number of cases ranged from a low of 2,928 in 2005 to as high as 10,952 in 2013 (25).

This study aimed to describe the genotypes of Ot in PCR-confirmed fever patients in Chiangrai and Tak provinces which border Myanmar (Chiangrai and Tak) and Laos (Chiangrai) with extensive cross-border population movements.

## 2 Materials and methods

### 2.1 Study setting

Chiangrai Prachanukroh Hospital is a tertiary-level government hospital located in Chiangrai province with approximately 800 inpatient beds, and roughly 4000 patients admitted monthly. The Shoklo Malaria Research Unit (SMRU) operates free-of-charge walk-in clinics for the underprivileged migrant community on the northwestern border of Thailand with Myanmar. Patients are invited to participate in clinical research designed to benefit the local population. Study participants are primarily agricultural workers from a rural tropical setting.

### 2.2 Ethics statement

This study was part of the Scrub Typhus Antibiotic Resistance Trial (START) and Eschar Investigations in Scrub Typhus (EXIST) study. START (Clinicaltrials.gov identifier NCT03083197) is a randomized controlled trial comparing azithromycin and doxycycline to treat non-severe scrub typhus in adults. In contrast, EXIST (Clinicaltrials.gov identifier NCT02915861) is an observational study evaluating the pathophysiology of scrub typhus in eschar biopsies and the diagnostic utility of eschar samples. START and EXIST studies were approved by the Chiangrai Hospital Ethics Committee (EC CRH 038/60 and CRH 044/59 Ex), Chiangrai, Thailand; the Faculty of Tropical Medicine Ethics Committee, Mahidol University, Bangkok (Ethics Reference: MUTN 2016-064-02 and MUTN 2016-079-01); the Oxford Tropical Research Ethics Committee, University of Oxford, UK (Ethic Reference: OxTREC 4-17 and 46-15). All patients provided written informed consent before sample collection. For underage subjects, written informed assent from participants and written informed consent from the participant’s parent(s)/legal guardian(s) were obtained.

### 2.3 Patient samples

Whole blood samples were collected from patients admitted at Chiangrai Pra-chanukroh Hospital (START and EXIST) and Shoklo Malaria Research Unit clinics (START) with a history of fever of 14 days or less, clinical suspicion of scrub typhus and a positive scrub typhus IgM RDT (InBios International, Inc. USA). For subjects who presented with an eschar lesion, scrapings were also obtained. From June 2018 to December 2019, patients with acute undifferentiated febrile illness (AUFI) were recruited as part of START and EXIST.

### 2.4 Nucleic acid extraction

Genomic DNA was extracted from the buffy coat and eschar (if present) samples using the DNeasy Blood & tissue kit (Qiagen, Germany). For each patient, where available, eschar samples were cut into pieces (~3 mm in diameter), and 200 µL of buffy coat was used for the DNA extraction following the manufacturer’s instructions. DNA was eluted in 50 µL AE elution buffer and then subjected to molecular testing.

### 2.5 Ot screening and 56kDa TSA amplification

Genomic DNA extracted from buffy coat and eschar were screened for the presence of Ot using a TaqMan real-time PCR assay targeting the 47kDa periplasmic serine protease *htrA* gene using the primer and the probe of OtsuFP630 for forward, OtsuRP747 for reverse primer and OtsuPR665 for probe (28).

Nested PCR further confirmed the positive samples from the real-time PCR screening. PCR amplification of 803 bp of 56 kDa TSA gene was carried out using primers previously described by Horinouchi et al. (1997) (29). In brief, primers RTS_8 and RTS_9 were used for the 1^st^ round of PCR, followed by the amplification of 650 bp of 56kDa TSA using RTS_6 and RTS_7 primers in the 2^nd^ round of PCR. The PCR conditions used were as follows: PCR was performed in 25 µL of a sample, consisting of 1 µM of each 56kDa TSA forward and reverse primer, 200 µM of each deoxynucleotide triphosphate, 2.5 U of GoTaq® DNA polymerase (Promega Corp., Madison, WI, USA), 1X PCR buffer, and 1 µL of DNA template. All samples were tested in duplicate. DNA fragments were pre-denatured at 95°C for 3 min, followed by 30 cycles of denaturation at 94°C for 1 min, annealing at 55°C for 1 min, extension at 72°C for 1 min, and post-extension at 72°C for 5 min (T100 thermal cycler, Biorad). The oligonucleotide sequences are as follows:

OtsuFP630: 5’-AACTGATTTTATTCAAACTAATGCTGCT-3’

Ots u RP747: 5’-TATGCCTGAGTAAGATACRTGA ATRGAATT-3’

OtsuPR665: 5’-6-FAM-TGGGTAGCTTTGGTGGACCGATG TTTAATCT-BHQ1-3’

RTS_6: 5’-GTTGGAGGAATGATTACTGG-3’

RTS_7: 5’-AGCGCTAGGTTTATTAGCAT-3’

RTS_8: 5’-AGGATTAGAGTGTGGTCCTT-3’

RTS_9: 5’-ACAGATGCACTATTAGGCAA-3’

Gel electrophoresis was performed with a 1.5% agarose gel electrophoresis in TBE buffer stained with 2.5% RedSafe™ (iNtRON Biotechnology, Inc., Seongnam, Gyeonggi-do, Korea). The gel ran at 100 volts for 50 minutes. The DNA band was visualized and photographed under UV light, and products were compared to the positive control sample and a known molecular size marker (EZload, Biorad Co, UK).

### 2.6 Sequencing and phylogenetic analysis

PCR-positive samples were purified, and the sequences were obtained by the Sanger sequencing method (Macrogen Inc. Seoul, South Korea). The nucleotide basic local alignment search tool (BLASTn) (30) was used to compare the DNA sequences obtained in this study with those on the Genbank database. Multiple sequence alignment was performed using Bioedit version 7.2.5 (31), and a phylogenetic tree was reconstructed with the maximum likelihood (ML) method using the MEGA 7 program on the Tamura 3-parameter model (32). Bootstrap analysis with 1000 repetitions was performed to test the robustness of the tree branching.

## 3 Results

### 3.1 Ot screening and 56kDa TSA amplification

Of 31 febrile patients testing positive for scrub typhus by scrub typhus IgM RDT, 18 were confirmed Ot-infected by PCR assay and underwent 56kDa TSA sequencing. One sample was rejected as the sequencing results showed mixed sequences, resulting in a final total of 17 patient samples analyzed for this study.

The 17 patient samples with Ot infection were collected from June 2018 to December 2019. Eight patients were from the EXIST study; nine were from the START study, and one with antibiotic treatment failure. A total of twenty-five samples were sequenced from the 17 patients, including 8 patients where both eschar and buffy coat were sequenced.

All patients had tested positive by scrub typhus IgM RDT. Of the 17 patient samples, 16 (94%) buffy coat and 11 (100%) eschar lesions samples were confirmed positive by 47kDa real-time PCR and 56kDa TSA nested PCR (Table 1).

### 3.2 PCR and DNA sequencing

**Figure 1:**
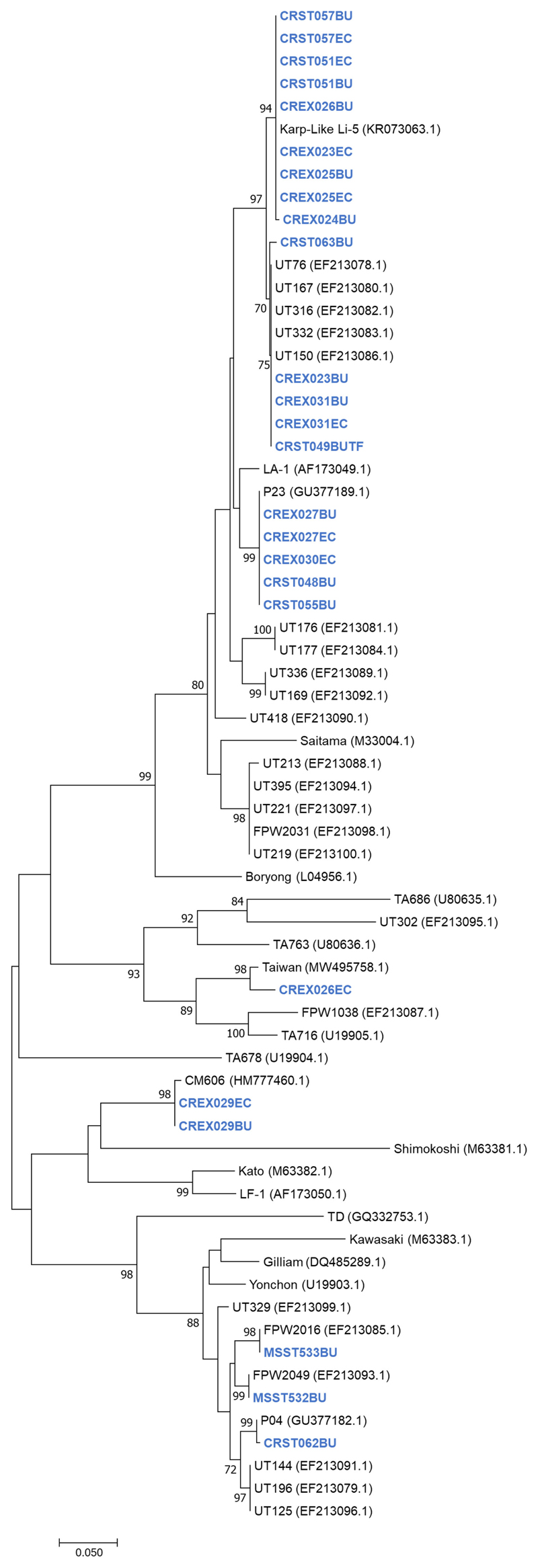
Unrooted phylogenetic reconstruction of the partial 56kDa TSA gene using the maximum likelihood method based on the Tamura 3-parameter model. The tree topology is drawn to scale, with branch lengths indicating the number of substitutions per site. Bootstrap values higher than 70% are indicated. Samples sequenced in this study are in blue bold.

A phylogenetic tree of the 56-kDa sequences obtained during this study and reference samples from Genbank showed that the sequences from Thailand clustered with the Karp, Gilliam, CM606, P23, P04 and Taiwanese MW495758.1 strains (Figure 1).

**Figure 2:**
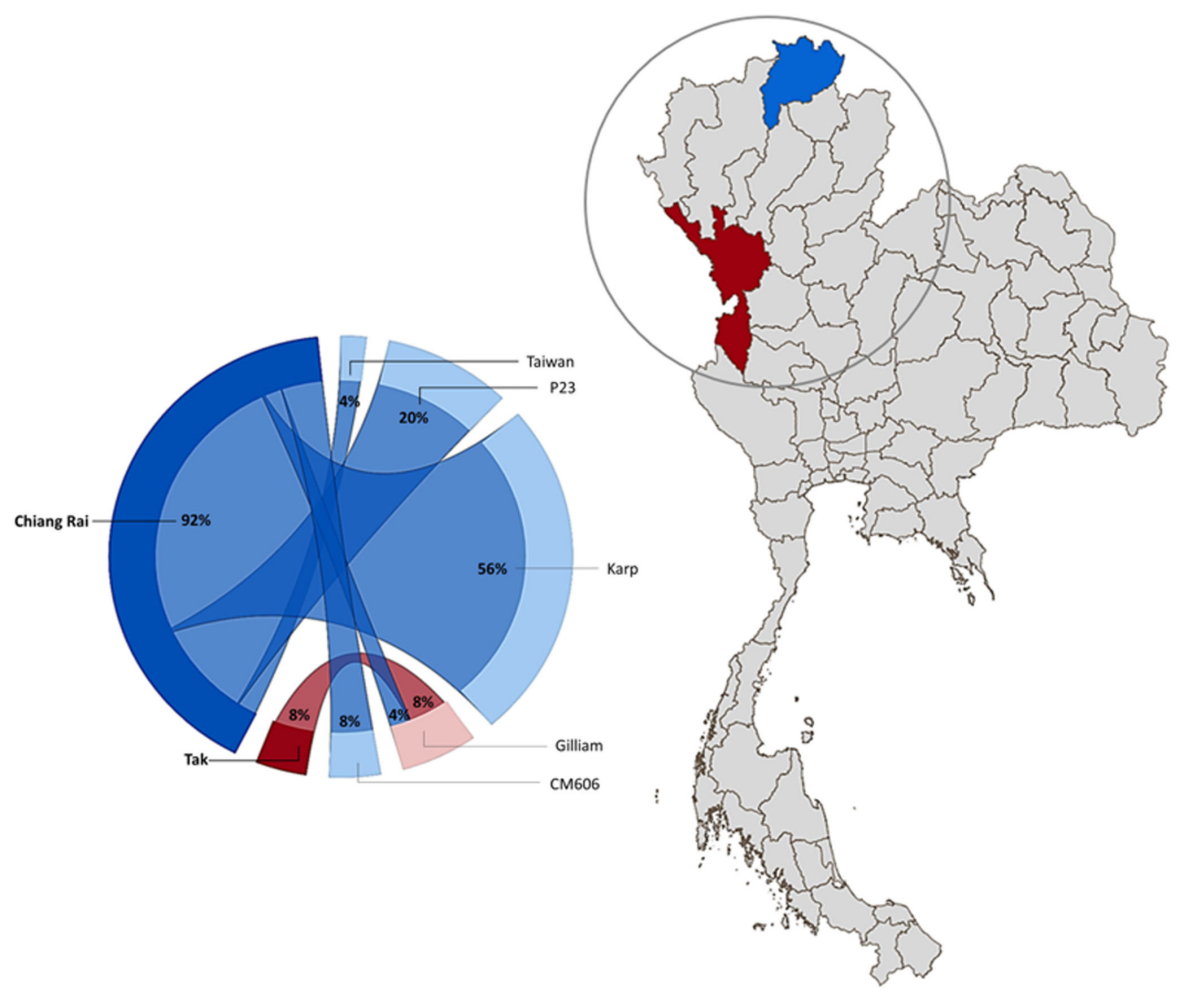
Geographical distribution of *O. tsutsugamushi* genotypes in Chiang Rai (blue) and Tak (Maroon) Province, Thailand. The circle diagram represents the percentage of Ot strains found in the study area (created using Power BI desktop).

Nucleotide blast results show nine nucleotide sequence samples (CREX023EC, CREX024BU, CRST051BU, CRST051EC, CREX025BU, CREX025EC, CREX026BU, CRST057BU, CRST057EC) demonstrated 99-100% sequence similarity to Ot Karp-like strain Li-5 (KR073063.1). Five nucleotide sequence samples (CREX023BU, CREX031BU, CREX031EC, CRST049BUTF, and CRST063BU) are closely related to the UT76 strain (EF213078.1) with 99.5-100% sequence similarity. Another five samples (CREX027BU, CREX027EC, CREX030EC, CRST048BU, and CRST055BU) had 100% identity to strain P23 (GU377189.1). Two nucleotide samples from one patient (CREX029; buffy coat and eschar crust) were identical and had 100% sequence similarity to CM606 (HM777460.1). In the next group, three samples were clustered in the same large cluster with the Gilliam reference strain (DQ485289.1), with each sample (CRST062BU, MSST532BU, and MSST533BU) most closely related to P04 (GU377182.1), FPW2016 (EF213085.1), and FPW2049 (EF213093.1) respectively, with 99.5-100% sequence similarity. A paired sample of buffy coat and eschar crust obtained from the same patient (CREX026) had different strains: the buffy coat strain belonged to the Karp group, while the eschar sample was closely related to 98.6% sequence similarity with the Taiwan strain (MW495758.1) (Figure 2).

## 4 Discussion

In this study, we report the genetic characterization of PCR-confirmed Ot-patients admitted to Chiangrai Prachanukroh Hospital and Shoklo Malaria Research Unit clinics with a history of fever of 14 days or less, clinical suspicion of scrub typhus, and a positive scrub typhus IgM RDT. Sequences of the 56kDa gene from 56% of all patients in this study were closely related to the Karp genotype, which agrees with past observations that Karp-like strains of Ot are predominant in Thailand (33, 34). We also found Taiwan-and Gilliam-like genotypes in this study. Strains related to CM606 (26) and P23 (5), first reported in Chiangmai, were also observed in this study (Figure 1). Further investigation into the distribution of these strains in northern Thailand, where Chiangrai and Chiangmai are located, is planned.

Previous studies of Ot in Thailand have reported a high diversity of genotypes. Wongprompitak et al. examined patient samples from two time periods (2001-2003 and 2009-2010) across six provinces in Thailand and reported the Karp, Kato/TA716, and JG-v (Japanese Gilliam variant) genotypes (34). In 2011, Ruangareerate et al. reported the Kato genotype of Ot and the different SEA1-3, LA, and TH1-2 strains (26). Blacksell et al. also documented the Karp, Gilliam, TA716 and TA763 type strains from patient samples collected between 2003 to 2005 in North East and Western Thailand (33). In 2013, Jiang et al. reported Karp, Kato, and Gilliam genotypes from the rodents and chiggers in Chonburi province of eastern Thailand (27). It is believed that the high genetic diversity could be an of the high migration rate within Thailand for work, education, and trade (35).

The CM606 strain was previously reported in Chiangmai province from patients’ blood and is distantly related to the Kato and LF-1 genotypes (26). For the current study, we found that one sample was closely related to CM606. The nucleotide sequences of five isolates were grouped with P23, also previously reported in 2013 in Chiangmai (5). P23 is also closely phylogenetically-associated with LA1, commonly found in Southeast Asia (26, 36).

In Vietnam, from 2015-2016, 67% of clinically suspected scrub typhus patients were confirmed PCR positive for scrub typhus, and Karp-like strains are the most common genotype in these patients (37). In Cambodia, between 2008 and 2010, scrub typhus patients at Calmette and Kantha Bopha hospitals reported that 43.5% of eschar and blood samples were of Karp-like genotypes (38). Serum samples from clinically suspected scrub typhus patients in Myanmar were sequenced using the 56 kDa TSA gene and reported 55.5% were Karp-like strains. The remaining samples were divided between Kato-, Gilliam-, and JG_C-like strains (39).

Phetsouvanh et al. reported that 8.6% of scrub typhus cases in Laos patients were infected with multiple Ot strains (40). In this study, one patient had a mixed infection with different strains detected in the buffy coat and eschar sample. Also, one sample was excluded because of mixed sequences in the plasma. Mixed infections may arise from a bite of a mite harboring more than one strain of Ot or multiple bites from mites harboring different strains that live in the same area (41–43). The clinical implication of mixed infections with different OT strains remains undetermined.

Of 31 febrile patients testing positive for scrub typhus by scrub typhus IgM RDT, 18 were confirmed Ot-infected by PCR assay, while 13 were negative. A negative result of PCR might be related to IgM RDT false positivity (44) or cross-reactivity with other species. Also, the scrub Typhus IgM RDT test will only indicate the presence of antibodies to Ot antigens in the specimen and may not imply active infection found by molecular detection.

An explanation for the discrepancy between blood and eschar OT genotypes may be related to the differences in infectivity and, thus, the virulence of each strain (45, 46). The more infective (and likely virulent) strain may be more likely to cause invasive infection than less infective strains. This remains a hypothesis supported by limited *in vitro* data (47).

The diverse 56kDa genotypes described in this study, and the finding that many strains are closely related to those found in previous studies, demonstrate that the diversity of Ot genotypes can be maintained long-term in mite populations. This has implications for vaccine research and development, as vaccines targeting multiple strains will be necessary to ensure vaccine efficacy and the development of appropriate diagnostics for that particular region and population.

The patient’s buffy coat, rich in white blood cells (48), continues to be the ideal choice for molecular detection, consistently giving a high positive when compared to whole blood (49). Eschar samples remain the favored diagnostic biological sample for scrub typhus detection (7). As noted in this study, 100% of eschar samples tested positive by PCR assay. This study confirms the utility of buffy coat and eschar samples for molecular diagnostics for scrub typhus. Additionally, the detection of Ot genomic material llows genotyping and the analysis of geographic Ot strain diversity, which, in turn, can benefit region-specific vaccine and diagnostic development.

This study has a few limitations. Only patient samples that were positive by scrub typhus IgM RDT, qPCR and nested PCR-positive were included in the study. The nucleotide sequencing results were based exclusively on partial 56kDa TSA (50). Further studies encompassing a wider geographical area and other significant genes are required better to understand the molecular epidemiology of scrub typhus in Thailand.

This study demonstrates the diversity of Ot genotypes in Chiangrai and Tak provinces, Thailand. We found the predominant strains were Karp-like, and we also detected Gilliam-, P23-, CM606-, and Taiwan-like strains. We also report the detection of CM606 and P23-like strains in Chiangrai patients, first reported in Chiangmai in 2010 and 2013, respectively. This finding indicates that strains of this type are potentially widely distributed in the northern part of Thailand that, might belong to the area with extensive cross-border population movement of humans and animals (35), and warrants further investigation. Molecular assays are helpful for diagnosis and improve our understanding of the Ot strain diversity and epidemiology. Ot strain diversity is once again shown even within a limited geographic region. We confirmed that Karp-like Ot strains dominate in northern Thailand, although other strains are present. The clinical significance of mixed infection with different Ot strains remains to be determined.

## Figures and Tables

**Table 1:** Molecular result of *O. tsutsugamushi*-suspected patient samples.

Sample number	Buffy coat		Eschar crust	
	47kDa qPCR	56kDa nPCR	47kDa qPCR	56kDa nPCR
CREX023	+	+	+	+
CREX024	+	+	+	+
CREX025	+	+	+	+
CREX026	+	+	+	+
CREX027	+	+	+	+
CREX029	+	+	+	+
CREX030	–	–	+	+
CREX031	+	+	+	+
CRST048	+	+	n/p	n/p
CRST049-TF	+	+	n/p	n/p
CRST051	+	+	+	+
CRST055	+	+	+	+
CRST057	+	+	+	+
CRST062	+	+	n/p	n/p
CRST063	+	+	n/p	n/p
MSST532	+	+	n/p	n/p
MSST533	+	+	n/p	n/p

+, positive result; -, negative; TF, treatment failed; qPCR, quantitative PCR; nPCR, nested PCR. n/p, eschar not present.

## Data Availability

The datasets presented in this study can be found in online repositories. The names of the repository/repositories and accession number(s) can be found below: https://www.ncbi.nlm.nih.gov/genbank/, OP548058-OP548082.
